# Silversol^®^ (a Colloidal Nanosilver Formulation) Inhibits Growth of Antibiotic-Resistant *Staphylococcus aureus* by Disrupting Its Physiology in Multiple Ways

**DOI:** 10.3390/pharmaceutics16060726

**Published:** 2024-05-28

**Authors:** Nidhi Thakkar, Gemini Gajera, Dilip Mehta, Vijay Kothari

**Affiliations:** 1 Institute of Science, Nirma University, Ahmedabad 382481, India; 21ftphds68@nirmauni.ac.in (N.T.); 20ftphds55@nirmauni.ac.in (G.G.); 2 Viridis BioPharma Pvt. Ltd., Mumbai 400043, India; dilipmehta39@hotmail.com

**Keywords:** antibiotic resistance, arginine biosynthesis, biofilm, efflux, membrane permeability, nanosilver, network analysis, transcriptome

## Abstract

Antibiotic-resistant strains of *Staphylococcus aureus* are being viewed as a serious threat by various public health agencies. Identifying novel targets in this important pathogen is crucial to the development of new effective antibacterial formulations. We investigated the antibacterial effect of a colloidal nanosilver formulation, Silversol^®^, against an antibiotic-resistant strain of *S. aureus* using appropriate in vitro assays. Moreover, we deciphered the molecular mechanisms underlying this formulation’s anti-*S. aureus* activity using whole transcriptome analysis. Lower concentrations of the test formulation exerted a bacteriostatic effect against this pathogen, and higher concentrations exerted a bactericidal effect. Silversol^®^ at sub-lethal concentration was found to disturb multiple physiological traits of *S. aureus* such as growth, antibiotic susceptibility, membrane permeability, efflux, protein synthesis and export, biofilm and exopolysaccharide production, etc. Transcriptome data revealed that the genes coding for transcriptional regulators, efflux machinery, transferases, β-lactam resistance, oxidoreductases, metal homeostasis, virulence factors, and arginine biosynthesis are expressed differently under the influence of the test formulation. Genes (*argG* and *argH*) involved in arginine biosynthesis emerged among the major targets of Silversol^®’^s antibacterial activity against *S. aureus*.

## 1. Introduction

Historically, silver has long been in use for various therapeutic purposes. It is an important ingredient of multiple traditional medicine formulations [1,2,3]. Modern times have also witnessed numerous reports [4,5,6] describing different types of biological activities of silver. Antimicrobial activity of different forms of silver against different groups of microorganisms has also been reported, e.g., antiviral [7,8,9], antibacterial [7,10,11], antifungal [12,13], antiprotozoal [14], and anthelmintic [15,16]. Different forms of silver (e.g., silver salts, metallic, or colloidal) may have different mechanisms of action and varying magnitudes of biological efficacy. The antibacterial potency of silver assumes importance in the face of the AMR (antimicrobial resistance) pandemic. A review of the literature did not provide strong indications of bacteria developing resistance against silver [17]. Silver is reported to exert its bactericidal activity by modulating cell membrane permeability, interrupting DNA replication, and inducing the generation of reactive oxygen species (ROS) [18,19,20,21,22]. However, a more detailed investigation is still required to reveal additional molecular mechanisms underlying the antibacterial activity of silver.

We chose a colloidal nanosilver solution as a test formulation for this study, as the metallic form of silver is believed to be more potent than its ionic counterpart [23]. Nanoparticles are defined as particles of size < 100 nm [24], and nanoparticles of metals can be expected to score high on the parameter of bioavailability compared to larger particles of the same metal [25]. The potential of metal oxide nanocarriers for targeted drug delivery toward the treatment of infectious diseases and as biomedical materials has been nicely reviewed by Saha et al. [26] and Nikolova and Chavali [27], respectively. Due to their small size and larger surface area, nanoparticles show enhanced colloidal stability and, therefore, increased bioavailability.

The target pathogen for this study was an antibiotic-resistant strain of *Staphylococcus aureus*. This Gram-positive bacterium is amongst the few most well-known and versatile bacterial pathogens, whose antibiotic-resistant phenotypes are listed by various public health agencies like the World Health Organization (WHO; https://www.who.int/publications/i/item/WHO-EMP-IAU-2017.12; accessed on 23 April 2024), Centers for Disease Control and Prevention (CDC; https://www.cdc.gov/drugresistance/biggest-threats.html; accessed on 19 April 2024), and the Indian government’s Department of Biotechnology (DBT; https://dbtindia.gov.in/sites/default/files/IPPL_final.pdf; accessed on 29 March 2024); moreover, this bacterium is among the priority pathogens for which there is a pressing requirement to discover new antibacterial agents [28]. The present study examined the effect of Silversol^®^ (a colloidal nanosilver formulation) on *S. aureus*’s growth, various important phenotypic traits, and gene expression profile at the whole transcriptome level. Silversol already has a history of clinical use for the management of oral health [29], skin diseases, diabetic ulcers, burns, and wound care/wound disinfection (https://www.rxsilver.com/index_htm_files/ABLSilversafety.pdf; accessed on 29 March 2024). As *S. aureus* is one of the most common clinical isolates [30,31], we made an effort to gain insights into Silversol’s antibacterial activity against this particular pathogen.

## 2. Materials and Methods

### 2.1. Colloidal Silver Formulation

The Silversol^®^ test formulation (100 ppm; batch ID: V-Silwater22-47), originally formulated by American Biotech Labs (American Fork, UT, USA), was sourced from Viridis BioPharma Pvt. Ltd., Govandi Gaonthan, Mumbai, India. This colloidal silver mixture is documented to exert various biological effects [23]. It comprises zero-valent metallic silver particles in their elemental form that are covered in silver oxide. The manufacturer claims its particle size range to lie between 5 and 50 nm.

### 2.2. Bacterial Culture

The strain of *S. aureus* utilized in this study (ATCC 43300) was procured from HiMedia, Mumbai, India. Though this strain is claimed to be resistant to methicillin and oxacillin (https://www.atcc.org/products/43300; accessed on 1 March 2024), disc diffusion assay in our lab did yield a zone of inhibition of 26.5 ± 9.19 and 23 ± 7.07 mm surrounding methicillin and oxacillin discs (Table S1 and Figure S1). However, our transcriptome of this strain, conducted as a part of this study, showed this strain to be positive for expression of the *mecA* gene, whose presence is considered a defining characteristic for labeling *S. aureus* as methicillin-resistant [32]. Antibiogram of this strain generated in our lab showed it to be resistant to four macrolide antibiotics (erythromycin, clindamycin, azithromycin, and clarithromycin), and intermediate to amoxicillin/clavulanic acid. To characterize the test strain in terms of virulence, we challenged the nematode worm *Caenorhabditis elegans* with it (OD_764_ = 1.50), and it was able to kill 100% of worms within 24 h (Figure S2). In summary, the *S. aureus* strain used in this study can be said to possess the traits of antibiotic resistance and virulence.

This bacterium was maintained on Tryptone soy agar (HiMedia) slants. For the purpose of different assays described in this report, (unless described otherwise) it was grown in tryptone soy broth (pH 7.0 ± 0.2; HiMedia) for 24 ± 1 h at 35 °C. Inoculum was standardized as OD_625_ between 0.08–0.10 (at par with McFarland turbidity standard 0.5).

### 2.3. Growth Inhibition Assay

The effect of Silversol on bacterial growth was assessed using a broth dilution assay. *S. aureus* was inoculated (at 10% *v*/*v*) in tryptone soy broth with or without Silversol (0.1–60 ppm), followed by incubation at 35 °C for 24 ± 1 h. The total volume of the system was 2 mL in 15 mL test tubes. Random intermittent shaking was provided during incubation. At the end of incubation, cell density was measured as A_764_ (Agilent Cary 60 UV–Vis; Agilent, Santa Clara, CA, USA).

The minimum concentration of Silversol inhibiting ≥80% growth was considered as the minimum inhibitory concentration (MIC) [33]. From each of the tubes with no visible growth, 0.1 mL of culture broth was spread on tryptone soy agar plates. These agar plates were inspected for the appearance of bacterial colonies during a 72 h incubation (at 35 °C) to determine the minimum bactericidal concentration (MBC). Extended incubation for a total of 72 h was practiced to enable differentiation of the genuine bactericidal effect from any possible post-antibiotic effect [34,35]. The minimum concentration of Silversol, capable of inhibiting the appearance of bacterial growth on agar surfaces almost completely, was considered as the MBC. Vancomycin was used as a positive control.

The growth curve of the test bacterium in the absence and presence of Silversol (at the sub-MIC level) was also generated, wherein incubation was carried out under continuous shaking (120 rpm).

### 2.4. Antibiotic Susceptibility Assay

Antibiogram of Silversol pre-exposed-*S. aureus* was obtained using a disc diffusion assay performed in accordance with the Clinical and Laboratory Standards Institute (CLSI) guidelines [36] and was compared with that of the control culture (which received no Silversol pre-exposure). *S. aureus* was grown in tryptone soy broth, with or without Silversol. Following overnight incubation (24 ± 1 h), 1 mL of culture broth was used for cell density quantification at 764 nm, and cells from the remaining 1 mL culture were pelleted down using centrifugation (10 min at 13,600× *g*). The pellet thus obtained was then resuspended in 1 mL of phosphate buffer (HiMedia; pH 7.0 ± 0.2), followed by centrifugation. The resulting cell pellet was utilized to prepare the inoculum for the subsequent disc diffusion assay by resuspending it in normal saline. The OD_625_ of the inoculum was adjusted to range from 0.08–0.10 to achieve equivalence to McFarland turbidity standard 0.5. The known volume (0.1 mL) of this inoculum was then spread onto cation-adjusted Muller–Hinton agar (HiMedia) in 150 mm plates (Borosil, Mumbai, India), followed by loading antibiotic discs (Icosa G-I PLUS; HiMedia, Mumbai, India) onto the agar surface. The plates were then incubated at 35 °C for 18 ± 1 h, followed by the observation and measurement of the diameter of the zone of inhibition.

### 2.5. Biofilm Assay

**Figure 1:**
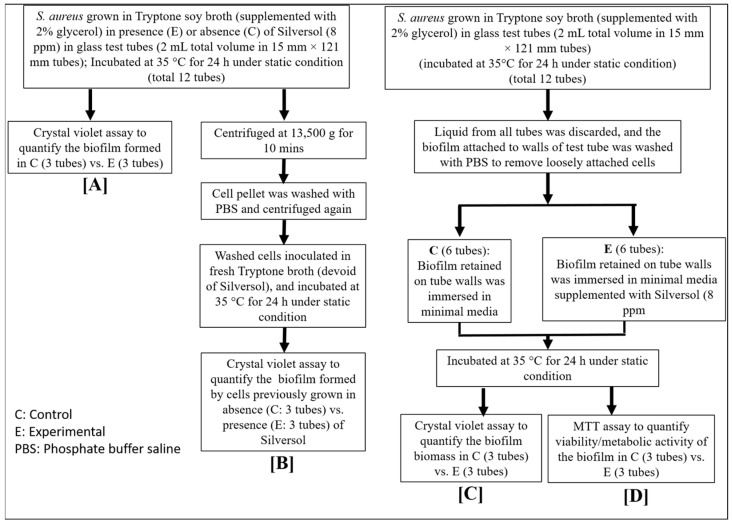
Flowchart depicting schematic of all biofilm assays. (**A**) Quantification of biofilm formation in the presence or absence of Silversol; (**B**) quantification of biofilm formation by silver-pre-treated vs. non-pre-treated *S. aureus* cells; (**C**) quantification of biofilm eradication after adding Silversol onto pre-formed biofilm; and (**D**) quantification of Silversol’s effect on metabolic activity of pre-formed biofilm.

Biofilm formation is an important virulence trait, and hence the effect of Silversol on the biofilm-forming ability of *S. aureus*, as well as on pre-formed biofilm was investigated using four different biofilm assays using the methodology described in our previous publication [37]. A flowchart illustrating all the biofilm assays is provided in Figure 1, wherein biofilm quantification was carried out using crystal violet assay [38], and biofilm viability assessment was conducted using MTT assay [39]. Vancomycin at the sub-MIC level (3 ppm) was used as a positive control.

For the crystal violet assay, the biofilm-containing tubes (after discarding the inside liquid) were subjected to washing with phosphate buffer saline (PBS) to remove all the non-adhering planktonic bacteria and then air-dried for fifteen minutes. Then, each of the washed tubes was subjected to staining with 2 mL of 0.4% aqueous crystal violet (Central Drug House, Delhi, India) for thirty minutes. Each tube was then washed twice with sterile distilled water (2 mL), followed by immediate de-staining with 2 mL of 95% ethanol. After allowing de-staining for 45 min, 1 mL of de-staining solution was transferred into separate tubes, and A_580_ was measured (Agilent Cary 60 UV–Vis; Agilent, Santa Clara, CA, USA).

For the MTT assay, the tubes containing biofilm (after discarding the inside liquid content) were washed with PBS to remove all the non-adherent bacteria, and then air-dried for fifteen minutes. Then, 1.8 mL of minimal media (NaCl 1 g/L, K_2_HPO_4_ 5 g/L, MgSO_4_ 0.1 g/L, NH_4_Cl 2 g/L, sucrose 15 g/L, yeast extract 0.1 g/L, pH 7.4 ± 0.2) was added into each tube, followed by addition of 200 µL of 0.3% MTT [3-(4,5-Dimethylthiazol-2-yl)-2,5-iphenyltetrazolium Bromide; HiMedia]. Following a 24 h incubation at 35 °C, all liquid was discarded, and the remaining purple formazan derivatives were dissolved in 2 mL of DMSO, and then A_540_ was measured.

### 2.6. Exopolysaccharide (EPS) Quantification

*S. aureus* was grown in 20 mL of tryptone soy broth contained in 100 mL flasks (containing or not containing Silversol). Incubation was provided at 35 °C for 24 ± 1 h under static conditions with intermittent shaking. At the end of incubation, cell density was quantified as OD_764_. For EPS quantification [40], the culture broth was centrifuged (13,600× *g*; 10 min). To the resulting supernatant, chilled acetone (Merck, Mumbai, India) was added in a 1:2 ratio and allowed to rest for 30 min for the EPS to precipitate. Thus, the precipitated EPS was then separated out by filtration using pre-weighed Whatman #1 filter paper (Whatman International Ltd., Buckinghamshire, UK). Filter paper containing EPS was then dried for 24 h, and the weight of the EPS on the paper was calculated.

### 2.7. Efflux Assay

An ethidium bromide (EtBr) efflux assay with *S. aureus* was performed using the method described in [41]. *S. aureus* cells, grown overnight in tryptone soy broth medium, were loaded with 8 ppm of EtBr (HiMedia) in the presence of 75 ppm of reserpine (HiMedia; positive control) or Silversol (8 ppm). Any effective efflux inhibitor like reserpine will inhibit efflux during this loading step. Cells were incubated at 35 °C for 20 min and then pelleted by centrifugation (13,600× *g*; 10 min). The medium was decanted, and the cell pellet was resuspended in fresh tryptone soy broth medium, either with or without Silversol (8 ppm), to an optical density of OD_764_ = 0.20. The EtBr efflux was then determined by quantification of fluorescence at excitation and emission wavelengths of 530 and 600 nm in a spectrofluorometer (JASCO FP-6500; Jasco International Co. Ltd., Tokyo, Japan).

### 2.8. Protein Estimation

The extracellular protein present in the bacterial culture (grown in the presence or absence of Silversol) supernatant, and intracellular protein in the cell lysate was quantified using the Folin–Lowry method [42,43]. After measuring cell density, 1 mL of *S. aureus* culture was centrifuged (13,600× *g*), and the resulting supernatant liquid was utilized for extracellular protein estimation. The left cell pellet was subjected to lysis [44] for the release of intracellular proteins. Briefly, the cell pellet was subjected to washing with phosphate buffer (pH 7.4) and centrifugation (13,600× *g*). The resulting pellet was then reconstituted in 1 mL of chilled lysis buffer (0.1 g of sodium dodecyl sulfate, 0.5 g of sodium deoxycholate, 0.60 g of Tris HCl, 0.876 g of NaCl, and 1 mL of Triton X-100, in 99 mL of distilled water) and centrifuged (500 rpm) for 30 min at 4 °C to provide agitation. This was followed by another round of low-temp (4 °C) centrifugation (13,600× *g*) for 20 min. The resulting cell lysate (i.e., supernatant) was utilized for protein estimation.

### 2.9. Membrane Permeability Assay

A propidium iodide (PI) uptake assay [45,46] was performed to quantify membrane permeability. *S. aureus* cells grown overnight in tryptone soy broth were centrifuged, and the cell pellet was dissolved in PBS to attain OD_764_ = 1.50 ± 0.05. This cell suspension was exposed to Silversol (8 ppm) for 30 min at 35 °C in an incubator. The negative control contained cells with no Silversol exposure. After incubation, 2 ml of each test sample was centrifuged at 13,600× *g*. The resulting pellet was washed with PBS, resuspended in PBS, and then PI (Invitrogen, Mumbai, India) at a final concentration of 10 ppm was added into the suspension. This mixture was kept in the dark for 10 min, followed by quantification of fluorescence, employing 544 nm as excitation and 612 as emission wavelength, using a spectrofluorometer (JASCO FP-6500). Triton-X (final concentration 0.5% *v*/*v*) was kept as a positive control.

### 2.10. Transcriptome Analysis

To unravel the molecular mechanisms through which Silversol inhibited bacterial growth and modulated various physiological traits, the gene expression pattern of *S. aureus* challenged with the sub-MIC level of Silversol (8 ppm) was compared with that of the control culture at the whole transcriptome scale. The overall workflow of this whole transcriptome analysis (WTA) aimed at capturing a holistic picture regarding modes of action of the test formulation is given in Figure S3.

#### 2.10.1. RNA Extraction

Bacterial RNA was extracted by the Trizol (Invitrogen Bioservices, Mumbai, India; 343909) method. Precipitation was achieved by isopropanol followed by washing with ethanol (75%), and RNA was then dissolved in nuclease-free water. The extracted RNA was quantified on a Qubit 4.0 fluorometer (Thermofisher, Mumbai, India; Q33238) employing an RNA HS assay kit (Thermofisher; Q32851) as per the manufacturer’s protocol. The concentration and purity of RNA were assessed using Nanodrop 1000. Finally, the RIN (RNA Integrity Number) value was determined by checking the RNA on the TapeStation using HS RNA ScreenTape (Agilent) (Table S2).

#### 2.10.2. Library Preparation

Final libraries were quantified using a Qubit 4.0 fluorometer (Thermofisher; Q33238) using a DNA HS assay kit (Thermofisher; Q32851). To determine the insert size of the library, it was queried on Tapestation 4150 (Agilent) using highly sensitive D1000 screentapes (Agilent; 5067-5582). The acquired sizes of all libraries are given in Table S2.

#### 2.10.3. Genome Annotation and Functional Analysis

Quality assessment of the raw fastq reads of the sample was achieved using FastQC v.0.11.9 (default parameters) [47]. The raw fastq reads were pre-processed using Fastp v.0.20.1 [48], followed by a reassessment of the quality using FastQC.

The *S. aureus* genome (GCA_000006765.1_ASM676v1) was indexed using bowtie2-build [49] v2.4.2 (default parameters). The processed reads were mapped to the indexed *S. aureus* genome using bowtie2 v2.4.2 parameters. The aligned reads from each of the samples were quantified using feature count v. 0.46. 1 [50] to determine the gene counts. Gene counts thus obtained were then fed as inputs to edgeR [51] using an exact test (parameters: dispersion 0.1) for estimation of differential expression. The down- and up-regulated sequences were acquired from the *S. aureus* coding file and fed into blast2go [52] for annotation to generate the gene ontology (GO) terms. Latter terms were then processed using the Wego [53] tool to create gene ontology bar plots.

All the raw sequence data were submitted to the Sequence Read Archive. The relevant accession number is SRX15156375 (https://www.ncbi.nlm.nih.gov/sra/SRX15156375; accessed on 20 January 2024).

### 2.11. Network Analysis

From among all the differentially expressed genes (DEGs) in Silversol-exposed *S. aureus*, those fulfilling the dual filter criteria of False Discovery Rate (FDR) ≤ 0.05 and log fold change ≥ 2 were selected for further network analysis. A list of such DEGs was input into the STRING (v.11.5) database [54] to create the PPI (Protein–Protein Interaction) network. Members of this PPI network were then arranged in descending order of ‘node degree’ (a quantitative indication of connectivity with other proteins or genes), and those above a specified threshold value were forwarded for ranking by the cytoHubba plugin (v.3.9.1) [55] of Cytoscape [56]. As cytoHubba uses twelve different ranking methods, we considered the DEGs to be top-ranked by a minimum of 6 different methods (50% of the total ranking methods) for further investigation. These top-ranked proteins were then shortlisted for local cluster analysis using STRING, and those that were part of multiple clusters were labeled as potential ‘hubs’ to be investigated for additional validation of their targetability. The term ‘hub’ refers to a protein or gene interacting with multiple other proteins/genes. These identified hubs were then subjected to co-occurrence analysis across genomes of multiple pathogens to visualize whether an antibacterial agent targeting these hubs is likely to exert a broad-spectrum activity. The above-described sequence of operations enabled us to end up with a limited number of proteins fulfilling multiple biological and statistical significance criteria simultaneously: (i) FDR ≤ 0.05; (ii) log fold change ≥ 2; (iii) relatively higher node degree; (iv) top-ranking by a minimum of 6 cytoHubba methods; and (v) member of >1 local network cluster.

### 2.12. Polymerase Chain Reaction (RT-PCR)

Differential expression of the potential hubs found using network analysis of DEGs revealed from WTA was further confirmed using PCR. Primer designing for the shortlisted genes was carried out using Primer3 Plus [57]. These primer sequences were checked for their binding exclusivity to the target gene sequence within the whole *S. aureus* genome. Primer sequences for these target genes are given in Table 1. RNA extraction and quality checks were carried out in the same way as described in the preceding section. cDNA synthesis was carried out using the synthesis kit SuperScript™ VILO™ (Invitrogen Biosciences). PCR assay employed the gene-specific primers purchased from Sigma-Aldrich. *rpsU* was run as the endogenous control gene. FastStart Essential DNA Green Master mix (Roche, Darmstadt, Germany; 06402712001) was used as the reaction mix. The real-time PCR assay was performed on a QuantStudio 5 real-time PCR machine (Thermo Fisher Scientific, Waltham, MA, USA). The temperature profile followed is given in Table S3.

### 2.13. Statistics

All results reported are means of three or more independent experiments, each performed in triplicate. Statistical significance was assessed using a *t*-test performed using Microsoft Excel^®^ (Version 2108), and data with *p* ≤ 0.05 were considered to be significant.

## 3. Results and Discussion

### 3.1. Growth Inhibitory Effect of Silversol against S. aureus Follows a Non-Linear Dose–Response Pattern

**Figure 2:**
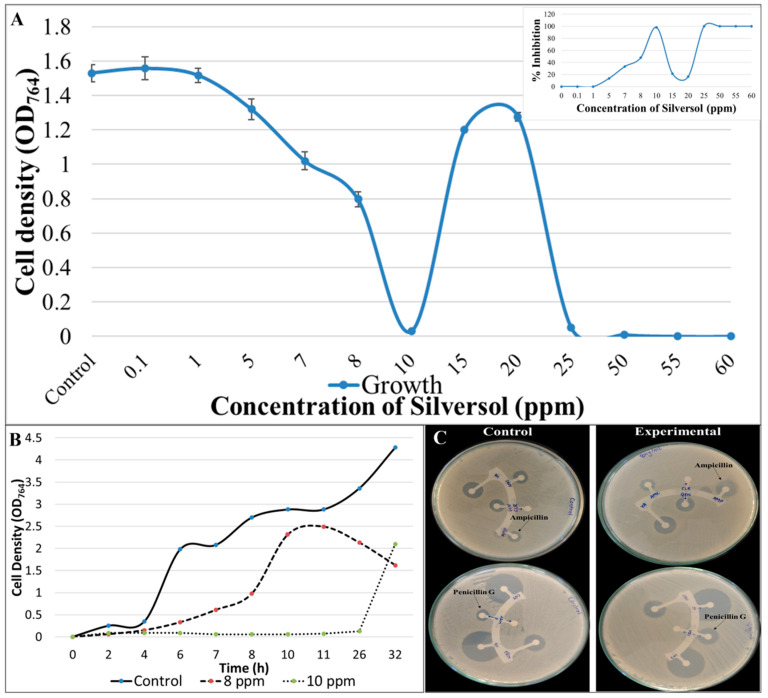
Silversol inhibits the growth of *S. aureus* and enhances its susceptibility toward β-lactams. (**A**) Silversol inhibits the growth of *S. aureus.* Bacterial growth was measured as OD_764_. Vancomycin (5 µg/mL) employed as the positive control inhibited bacterial growth by 100%. All the OD points plotted differed from the control at *p* < 0.01. The inset box shows the plotted percent inhibition vs. time for a better idea of the shape of the dose–response curve. (**B**) Growth curve of *S. aureus* in the presence or absence of Silversol. *S. aureus* could achieve a lesser growth rate and cell density in the presence of bacteriostatic concentrations of Silversol. (**C**) Silversol-pre-treatment enhances bacterial susceptibility to beta-lactam antibiotics. Silversol-pre-treated cells (experimental panel) can be seen surrounded by larger zones of inhibition encircling discs of penicillin and ampicillin.

Silversol exerted its growth inhibitory effect against *S. aureus* at concentrations ≥5 ppm (Figure 2A). Interestingly, the dose–response curve assumed different shapes over different concentration ranges (Figure 2A, inset box), considering ‘inhibition of growth’ as the ‘response’. For example, in the 1–10 ppm range, it followed a pattern following the threshold model, wherein 5 ppm was the minimum concentration required to generate the response. In the 7–15 ppm range, it assumed an ‘inverted U’ shape, while in the 10–25 ppm range, it assumed a ‘U’ shape. These different patterns of dose–response relationship can be appreciated better by looking at relevant explanations in [58,59], etc. The non-linear nature of this dose–response curve stemmed from the fact that some of the higher concentrations (15–20 ppm) were less effective at inhibiting bacterial growth than some of the lower concentrations (8–10 ppm) (Supplementary Materials, Figure S4). Such paradoxical antibacterial effect of bactericidal substances, often referred to as the ‘Eagle Effect’, has been reported in previous literature too, with *S. aureus* as well as other bacteria. As early as 1948, Eagle and Musselman [60] reported an in vitro paradoxical effect of penicillin against *S. aureus* and streptococci. Here, the paradox is that a decrease was observed, instead of an increase, in bactericidal activity at concentrations exceeding the MBC. A similar paradoxical effect was also observed in the case of *S. aureus* challenged with cefotaxime [61]. Such observations were also reported in studies investigating the recovery of bacteria from the toxic effects of penicillin [62]. *Streptococcus faecalis* and Group B hemolytic streptococci exposed briefly to higher concentrations of penicillin were reported to require less time to recover from the toxic effects of penicillin than when they were exposed to lower concentrations for an equal duration. Penicillin at the dose level of 200 mg/kg given to mice infected with Group B beta-hemolytic streptococci killed the pathogen more slowly than the lower dose of 3 mg/kg [63]. Though over the decades, there have been numerous reports of anomalous drug activity where the usual direct relationship between concentration of antibacterial compounds and inhibition of bacterial growth is not followed, detailed mechanistic explanations for such non-linear responses have not yet penetrated the conventional microbiology wisdom. Further, all such cases of a non-linear dose–response relationship cannot be explained by any single concept, e.g., ‘Eagle effect’, ‘hormesis’, or any other such term. While it is most likely that further such examples will continue to appear in the literature, as and when discovered, wider reporting and acceptance of such ‘unusual’ observations across the microbiological community is desired, and so are the efforts to unravel the underlying mechanistic details. One possible explanation for such non-linear responses may be that the bacteria exhibit a hormetic response in order to enhance their resilience when encountered with subinhibitory concentrations of antibacterial formulations or any other stressors [59]. Since these responses have been reported with multiple bacteria and different antibiotics [64,65,66,67], hormesis can be viewed as a generalized adaptive mechanism implemented by bacteria to strengthen their resistance against antibiotics. Adoption of such defensive responses may be driven by the ability of low doses of antibacterial substances to modulate the transcriptional activity of bacteria. 

The minimum concentration of Silversol required to achieve complete visible inhibition of bacterial growth was 10 ppm, and hence it can be considered as the MIC. However, the growth-inhibitory effect of Silversol up to 25 ppm can largely be said to be bacteriostatic, as the cells exposed to these concentrations when plated on fresh media (devoid of Silversol) were able to give rise to lawn growth (Figure S5). Concentrations of Silversol ≥50 ppm exerted a bactericidal effect, and the cells exposed to these concentrations when plated on fresh media (devoid of Silversol) could give rise only to a few isolated colonies. Based on observation of those plates, 60 ppm can be considered as the MBC. Non-cidal action of Silversol at lower concentrations was also confirmed in the growth curve experiment, wherein the bacterium was able to partially overcome the growth-inhibitory effect of Silversol if allowed extended incubation (Figure 2B). However, the maximum cell density achieved by *S. aureus* in the presence of 8 ppm and 10 ppm Silversol was 1.71 and 2.03-fold less than that of the control. The onset of growth was much delayed in the presence of 10 ppm Silversol. While the control culture was still in the exponential phase of growth at the 32nd hour of incubation, the death phase had already started in the experimental culture (8 ppm Silversol) by the 26th hour.

Silversol’s 8 ppm level was found to inhibit the growth of *S. aureus* by nearly 50%, and hence considering this as ~IC_50_, all further experiments (unless specified otherwise) were conducted at this concentration.

### 3.2. Silversol Pre-Treatment Renders S. aureus More Susceptible to Ampicillin and Penicillin G

When Silversol-pre-treated *S. aureus* cells were subjected to antibiotic susceptibility assay using the disc diffusion method, their susceptibility to ampicillin and penicillin was increased by approximately 1.5-fold (Table 2; Figure 2C). To nullify any possible role of the ‘post-antibiotic effect’, we confirmed that Silversol-pre-treated cells grow at the same rate as that of the control when transferred into fresh media (devoid of silver). This resistance-modifying activity of Silversol becomes important in light of the fact that strains of *S. aureus* exhibiting beta-lactam resistance have established a considerable ecological niche among human pathogens [68]. As approximately only 10% of *S. aureus* clinical isolates (in the United States) are susceptible to penicillin (https://www.cdc.gov/hai/settings/lab/lab_mrsa.html; accessed on 3 January 2024), resistance modifiers making the bacteria more susceptible to one or more beta-lactams can help widen the utility spectrum of classical antibiotics.

### 3.3. Silversol Inhibits Biofilm Formation and Renders Pre-Formed Biofilm Non-Viable

**Figure 3:**
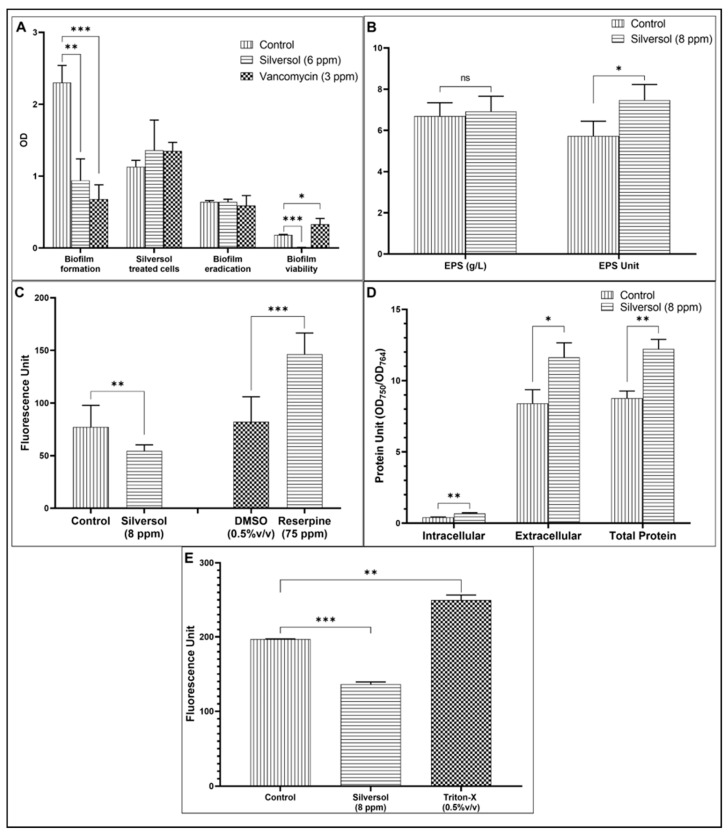
Silversol modulates various important physiological and phenotypic traits in *S. aureus*. (**A**) Silversol inhibits biofilm formation in *S. aureus* and renders pre-formed biofilm metabolically inactive. The non-inhibitory concentration of Silversol allowed *S. aureus* to form a lesser biofilm (first group of bars); Silversol-pre-treated cells formed biofilm at par to the control cells; when Silversol was added onto pre-formed biofilm, it was not able to eradicate it (third group of bars), but reduced its metabolic activity to a non-detectable level (fourth group of bars). Vancomycin employed as a positive control at the sub-MIC level inhibited growth by 26% ± 0.12 and biofilm formation by 70% ± 12.22. Vancomycin did not eradicate biofilm when added to pre-formed biofilm, but it increased its metabolic activity by 83% ± 30.8. While biofilm formation was quantified using crystal violet assay, biofilm viability was estimated using MTT assay. (**B**) Silversol enhances exopolysaccharide (EPS) synthesis in *S. aureus*. Though Silversol-supplemented media contained a lesser number of bacterial cells, they produced EPS in an amount at par with their Silversol-unexposed counterparts (first group of bars). However, when the EPS Unit was calculated as the cell density (OD_764_): EPS (g/L) ratio, this was found to be 30% ± 6 higher in the presence of Silversol. (**C**) Silversol triggers overexpression of efflux machinery in *S. aureus*. Fluorescence of the intracellularly accumulated EtBr was 29% ± 5.77 lesser in Silversol-exposed *S. aureus*. Higher (78% ± 20.12) intracellular accumulation of EtBr was observed in the presence of the known efflux inhibitor, reserpine. (**D**) *S. aureus* grown in the presence of Silversol exhibited higher protein synthesis and secretion. Extracellular and intracellular protein concentrations in *S. aureus* grown in the presence of Silversol at the sub-MIC level were significantly higher (38% ± 13.9 and 61 ± 12.7%, respectively) compared to its Silversol-non-exposed counterpart. Protein Unit (ratio of protein concentration: cell density) was calculated to rule out any effect of cell density on protein synthesis. (**E**) Silversol exposure reduces the membrane permeability of *S. aureus*. Silversol-pre-exposed cells allowed 30% ± 3.18 lesser propidium iodide (PI) to enter them. Cells pre-exposed to Triton-X, a known disruptor of bacterial membranes, allowed 26% ± 6.97 more PI to enter, as indicated by the fluorescence of intracellularly accumulated dye. * *p*  ≤  0.05, ** *p*  ≤  0.01, *** *p* ≤ 0.001; ns: not significant.

*S. aureus* in the presence of non-growth inhibitory concentration of Silversol could form 59% less biofilm than the control cells. Neither pre-treatment with Silversol could compromise the bacterial ability to form biofilm, nor could Silversol eradicate pre-formed biofilm. However, Silversol brought down the biofilm viability to zero when added to pre-formed biofilm (Figure 3A). These results indicate that Silversol could penetrate the biofilm matrix to exert its effect on the metabolic activity/viability of constituent cells. Reduced biofilm formation and decrease in biofilm viability in the presence of Silversol was also previously reported by us in the case of *Pseudomonas aeruginosa* [37]. This anti-biofilm activity of Silversol is important considering that bacterial biofilms exert antibiotic tolerance at higher levels, and biofilm infections are more difficult to treat [69]. Nanoparticles of antibacterial preparations have been demonstrated to possess notable efficacy against bacterial biofilms in a variety of bacterial species [70], and the results of the present study corroborate with the same. The anti-biofilm activity of Silversol can be one of the attributes of this formulation, making it effective at wound disinfection as biofilm-infected wounds take longer to heal [71] in the absence of an efficient anti-biofilm therapy.

### 3.4. Silversol Makes S. aureus Synthesize and Secrete More Exopolysaccharide (EPS)

EPS is an important component of the biofilm matrix, and it enables the bacterial pathogen to effectively adhere to the host and colonize there [72,73]. The EPS matrix can also protect the pathogen from a variety of challenges including host defense factors [74]. Silversol-pre-exposed *S. aureus* culture had a 1.30-fold higher EPS production (Figure 3B). Enhanced EPS secretion may be taken as a response to the stress induced in the bacterial population by Silversol, as EPS production is known to be a part of the bacterial stress response [75,76].

### 3.5. Silversol Triggers Overexpression of Efflux Machinery in S. aureus

*S. aureus* cells accumulated lesser EtBr when incubated with Silversol than when in Silversol-free media (Figure 3C). This lesser intracellular accumulation of EtBr can be said to have resulted from overexpression of efflux machinery in Silversol-exposed *S. aureus*. While efflux pumps play an important role in detoxification, their overexpression may have a negative effect on bacterial physiology [77], as overactive efflux machinery will export even some of the essential intracellular content. The results of this in vitro efflux assay corroborate well with the overexpression of efflux-associated genes (*terC* and *mepA*) in Silversol-exposed *S. aureus* described later.

### 3.6. Protein Synthesis and Export in S. aureus Is Enhanced in the Presence of Silversol

Extracellular and intracellular protein content in *S. aureus* culture grown in the presence of Silversol was found to be 1.38-fold and 1.61-fold higher, respectively, than the control (Figure 3D). This indicates that not only was overall protein synthesis increased but also its export was increased under the influence of Silversol. This increased protein export may have arisen from compromised membrane integrity and/or overexpression of efflux machinery [78,79,80]. Bacteria might be responding to the inhibitory effect of such antimicrobials acting as suppressors of protein synthesis by elevating their protein synthesis and/or secretion machinery to compensate for the inhibitory effect of the antibacterial substances [81]. Such up-regulation of protein synthesis can be believed to have originated from the translational reprogramming in the stressed bacterial cells [82] while they deal with the stress of Silversol’s antibacterial effect.

### 3.7. Silversol Alters Membrane Permeability of S. aureus

The membrane permeability of bacterial cells was quantified in terms of their ability to uptake the fluorescent dye propidium iodide. Lesser uptake of this dye by bacterial cells in the presence of Silversol indicates this formulation’s ability to alter membrane permeability (Figure 3E). Reduced permeability might have compromised bacterial capacity to allow intake of nutrients leading to stunted growth. This combined with overexpression of efflux function (Figure 3C) and protein secretion (Figure 3D) can be expected to force bacteria to face a scarcity of essential nutrients and metabolites. Alterations in membrane functioning can affect multiple bacterial traits such as surface charge, permeability, fluidity, stability of the bacterial membrane, antibiotic susceptibility, etc. [83]. A correlation between the antibacterial activity of Silversol and its ability to alter membrane fluidity cannot be ruled out, as expected of other membranotropic agents [84].

### 3.8. Silversol Causes Multiple Genes in S. aureus to Express Differentially

**Figure 4:**
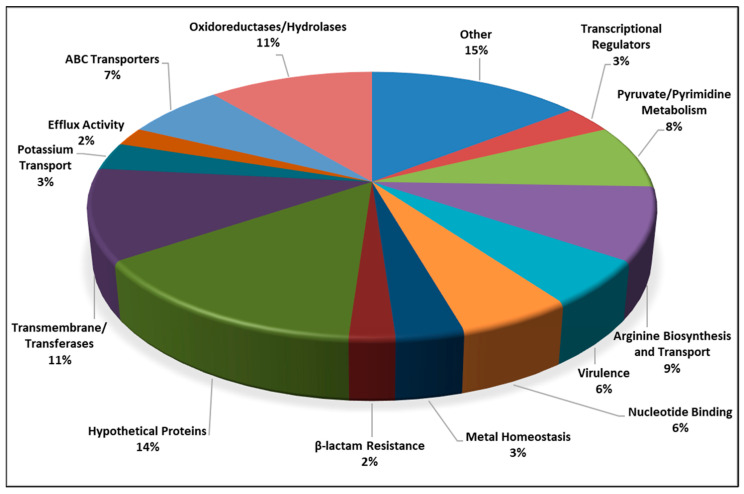
Function-wise categorization of the significantly differentially expressed genes in Silversol-treated *Staphylococcus aureus* (% values represent the fraction of the total number of DEGs).

After confirming the growth-inhibitory activity of Silversol against *S. aureus*, we compared the gene expression profile of the Silversol-exposed *S. aureus* with that of the control at the whole transcriptome level. Whole transcriptome analysis identified a total of 90 differentially expressed genes (3.32% of the total genome) in Silversol-treated *S. aureus*, of which 49 were down-regulated (Table 3) and 41 were up-regulated (Table 4). The corresponding heat map (Figure S6) and volcano plot (Figure S7) are provided in the Supplementary Materials. A summary of function-wise categorization of all the differentially expressed genes (DEGs) is presented in Figure 4.

Among the down-regulated DEGs, the one with the highest log fold change (logFC 5.14) value was *CidR,* which is the transcriptional regulator of the LysR family. This regulator is known to control stationary phase cell death in *S. aureus* [85] by influencing the expression of operons that display pro- and anti-death functions. Since the primary role of CidR regulon is to limit acetate-dependent potentiation of cell death in staphylococcal populations, its down-regulation can be expected to have a negative effect on bacterial growth. Besides regulating stationary-phase survival of *S. aureus*, the *CidR* gene also affects antibiotic tolerance in this bacterium [86].

Another heavily down-regulated gene in the Silversol-exposed *S. aureus* was *spa* (3.26↓) coding for staphylococcal protein A. The latter is a multifunctional virulence factor of *S. aureus*, which plays a role in the inhibition of the host innate and adaptive immune responses. Owing to its immunoglobulin-binding capacity, this protein confers protection on *S. aureus* from phagocytic killing via inhibition of the Ig Fc region. Additionally, the spa is known to prevent the host-elicited B-cell response, decrease long-term antibody production, inhibit osteogenesis, and act as a pro-inflammatory factor in the lung [87]. Protein A has also been identified as an essential component of the *S. aureus* biofilm, which induces bacterial aggregation in liquid medium and during biofilm formation. Besides interacting with multiple immunologically important eukaryotic receptors, protein A contributes considerably toward the development of biofilm-associated infections [88], and hence its down-regulation by Silversol can be of high clinical relevance.

In addition to the *spa*, other virulence-associated genes down-regulated in Silversol-exposed *S. aureus* were *TraH* (2.73↓) and the one coding for ABC transporter permease subunit (2.91↓). The latter is a part of the ABC transporter complex CntABCDF involved in the uptake of metal, particularly in the import of divalent metal ions like nickel, cobalt, and zinc. Its down-regulation may be expected to disturb metal homeostasis in bacterial populations as many important enzymes rely on their metal cofactors for proper functioning. This permease is also believed to contribute toward the virulence of *S. aureus*, as it has been shown to be necessary for full urease activity in vitro [89], and urease is well recognized as a virulence factor in multiple pathogenic bacteria [90,91,92].

TraH mentioned in the preceding paragraph is a conjugal transfer protein and a component of the Type IV secretion system (T4SS). Since for many Gram-positive pathogens, conjugative plasmid transfer is an important means of spreading antibiotic resistance, components of T4SS are being viewed as potential targets for alternative anti-pathogenic strategies [93].

Among other important gene clusters down-regulated in Silversol-exposed *S. aureus*, one notable cluster was the kdp system, which is a potassium uptake system. Three components [kdpA (2.62↓), kdpB (2.64↓), and kdpC (2.57↓)] of this system were found to be significantly down-regulated in our experimental culture. *kdpB* has been shown to be one of the core genes regulating cell death in *S. aureus* [94]. Potassium has many key functions within bacterial cells. For example, it is required for the activity of multiple intracellular enzymes, acts as an intracellular second messenger, and is involved in the maintenance of a constant internal pH and membrane potential. One of the clinically important characteristics of *S. aureus* is its ability to survive in/on high-salt areas of human nares and skin is also helped by its capacity for efficient potassium uptake and intracellular accumulation of this cation [95]. Since the kdp system is widely distributed among bacteria [96], any formulation targeting this system can be expected to exert a broad-spectrum antibacterial activity, and in fact, silver nanoparticles have been demonstrated to possess a broad activity spectrum in terms of their ability to inhibit the growth of different Gram-positive and Gram-negative bacteria [97,98].

Down-regulation of two of the genes, *mecA* and *blaZ*, associated with beta-lactam resistance corroborated well with results of antibiotic susceptibility (Figure 2C and Table 2), wherein Silversol-pre-exposure was found to increase the susceptibility of *S. aureus* to beta-lactams. The occurrence of *mecA* and *blaZ* genes has been reported in methicillin-resistant *S. aureus* associated with vaginitis among pregnant women [99]. Expression of these genes may be considered important for the expression of antibiotic-resistant phenotypes in clinical isolates, as the functionality of at least one *mecA* regulator is necessary for *S. aureus* to be oxacillin-resistant [100]. Penicillin resistance in *S. aureus* is manifested predominantly via the production of β-lactamase encoded by the *blaZ* gene, and testing for the presence of this gene is also recommended by the Clinical and Laboratory Standards Institute for cases of serious *S. aureus* infection [101].

Among the top ten up-regulated genes in Silversol-exposed *S. aureus*, two were *hrtA* (3.07↑) and *hrtB* (3.15↑). Four more genes (*vraA, vraC, vraD*, and *vraE*) coding for components of the ABC transporter complex hrt were also up-regulated. The HrtAB system is a hemin-regulated ABC transporter that protects *S. aureus* against hemin toxicity. Since *S. aureus* has been reported to launch an altered gene expression program involving differential expression of Hrt genes, when it senses membrane damage; differential up-regulation of *hrtA* and *hrtB* in our experimental culture can be taken as an indication of Silversol’s ability to alter membrane permeability in this bacterium. Overexpression of *hrtB* can lead to dysregulated pore formation, and its up-regulation has also been reported in *S. aureus* challenged with channel-forming antimicrobial peptides [102]. The altered abundance of Hrt proteins has also been reported in *S. aureus* cultures facing alterations in iron status [103], and hence up-regulation of these genes in the presence of Silversol can be considered an indication of this formulation’s ability to disturb iron homeostasis. Silversol’s potential ability to trigger iron starvation in *S. aureus* can be one of the reasons underlying its successful clinical applications (e.g., wound disinfection), as the role of bacterial iron acquisition during pathogenesis is well established [104,105].

Among the top 25 up-regulated genes in Silversol-exposed *S. aureus*, two [*terC* (2.63↑) and *mepA* (2.46↑)] were associated with efflux activity. Efflux machinery, besides throwing out toxic items, also has important physiological functions in bacteria [77]. In general, the up-regulation of efflux machinery can be taken as a sign of the bacteria making efforts for detoxification; for example, in the case of this study, they may be trying to efflux silver. However, overexpression of efflux function can lead to leakage of even essential molecules from within the cell, thereby negatively affecting bacterial growth and fitness. The way efflux inhibitors are considered potential anti-pathogenic agents [106], efflux agonists triggering overexpression of the efflux pumps can also be pursued as a novel antibacterial strategy. Of the two up-regulated genes mentioned above, TerC belongs to a family of integral membrane proteins and has been implicated in resistance to metal ions like tellurium [107] and Mn [108]. TerC helps bacteria alleviate metal toxicity by participating in the efflux of metal ions. In the case of the present study, TerC might have been activated in response to silver overload. Owing to the widespread occurrence of the TerC family proteins among bacteria and its possible influence on host–pathogen interactions, it can be an important antibacterial target [109,110]. Another efflux-associated up-regulated gene *mepA* is a multidrug resistance efflux protein involved in transporting several clinically relevant monovalent and divalent biocides, the fluoroquinolone antibiotics, norfloxacin and ciprofloxacin, and also tigecycline [111]. Since mepA has a broad substrate profile [112], its poorly regulated overexpression (as observed in Silversol-exposed *S. aureus*) can trigger the efflux of multiple substrates, probably including some essential metabolites. Though metal nanoparticles have been postulated to exert inhibitory action against multidrug resistance efflux pumps [113], their ability to trigger uncontrolled overexpression also needs to be investigated for possible clinical exploitation.

### 3.9. Network Analysis of DEGs

**Figure 5:**
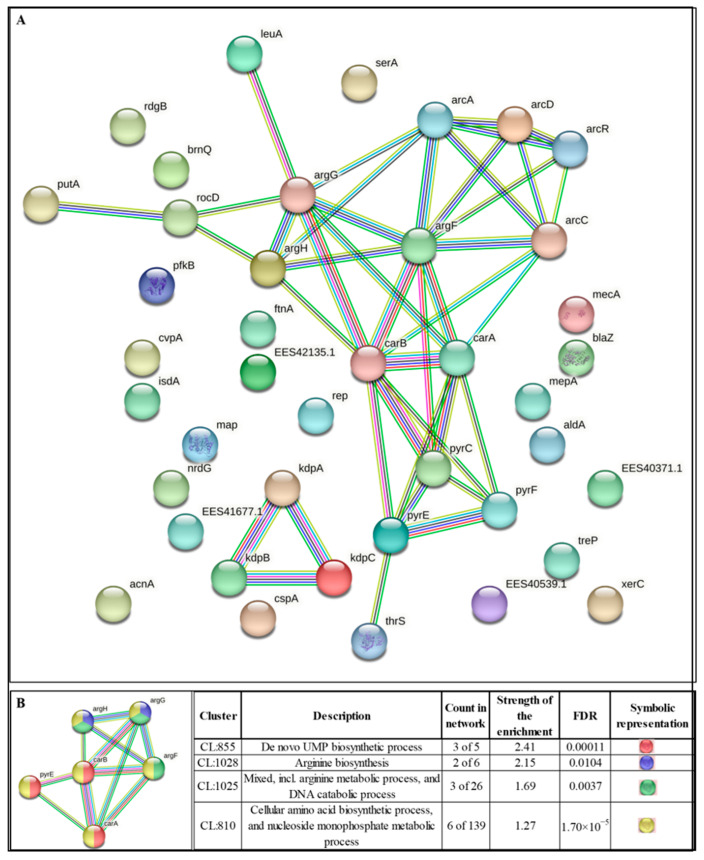
(**A**) Protein–Protein Interaction (PPI) network of DEGs following the dual criteria of fold change ≥ log 2 and FDR ≤ 0.05 in Silversol-exposed *S. aureus.* PPI enrichment *p*-value 3.81 × 10^−11^. (**B**) PPI network of top-ranked genes shortlisted using cytoHubba among DEGs in Silversol-treated *S. aureus.* PPI enrichment *p*-value 1.81 × 10^−14^.

**Figure 6:**
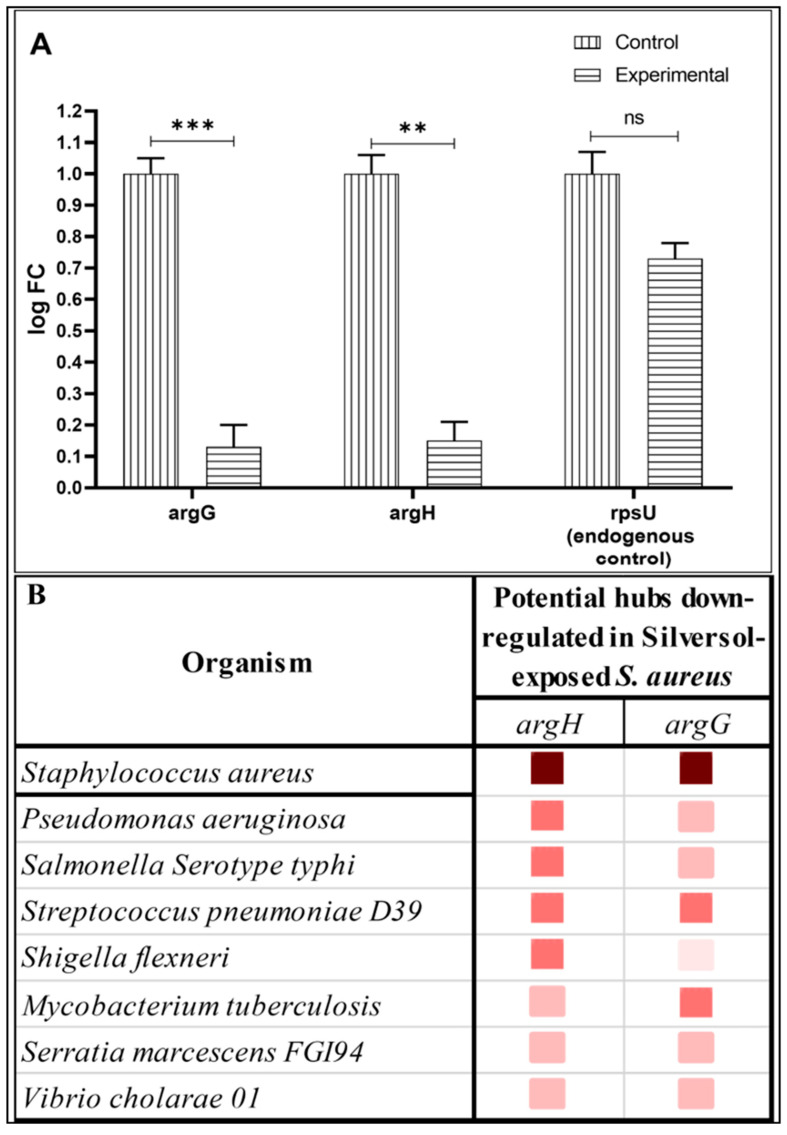
(**A**) Confirmation on differential expression of selected genes in Silversol-challenged *S. aureus* using RT-PCR. ** *p*  ≤  0.01, *** *p* ≤ 0.001, ns: not significant (**B**) Co-occurrence analysis of genes coding for potential targets in *S. aureus*, across multiple pathogens. The darker the shade of the squares, the higher the homology between the genes being compared.

To gain deeper insight into the interactions among DEGs identified in Silversol-exposed *S. aureus*, we subjected all the DEGs to network analysis using STRING. The resulting Protein–Protein Interaction (PPI) (Figure 5A) network had 41 nodes connected through 41 edges, with an average node degree of 2. Since the number of edges (41) in this PPI network is almost 3.4-fold higher than expected (12), this network can be believed to possess a significantly higher number of interactions among the member proteins than what can be expected for a random set of proteins having similar sample size and degree distribution. Such an enrichment is indicative of the member proteins being biologically connected, at least partially. When all the 41 nodes were arranged in descending order of node degree, 22 nodes appeared to have a non-zero score, and we shortlisted the top 13 genes (Table 3 and Table 4) with a node degree ≥ 3 to be forwarded for ranking by different cytoHubba methods (Table 5). Then, we checked for genes that were among the top-ranked candidates by ≥6 cytoHubba methods. This enabled the shortlisting of six genes, which were ranked among the top six by ≥9 cytoHubba methods to be subjected to further cluster analysis. The interaction map of these six important genes (Figure 5B) revealed them to be networked with an average node degree score of 4.33. The number of edges in this network was 13 compared to the expected 1, for any such random set of proteins. The six genes were distributed among three different local network clusters. Two of the genes, *argG* and *argH,* were part of two of these three clusters, hence we chose them for further RT-PCR validation. PCR assay did confirm the differential expression of these two genes in Silversol-exposed *S. aureus* (Figure 6A). Hence, arginine metabolism can be believed to be one of the major targets of Silversol in *S. aureus*.

All the six identified hubs were shown by cluster analysis to belong to amino acid biosynthesis (Figure 5B). Moreover, all six genes are participants in the arginine biosynthesis pathway. Three of them (*argG, argH*, and *argF*) are involved in the urea cycle, which is a part of the arginine biosynthesis pathway. Hence, amino acid metabolism in general, and arginine metabolism and urea cycle in particular, seem to be targeted by Silversol in *S. aureus*.

**Figure 7:**
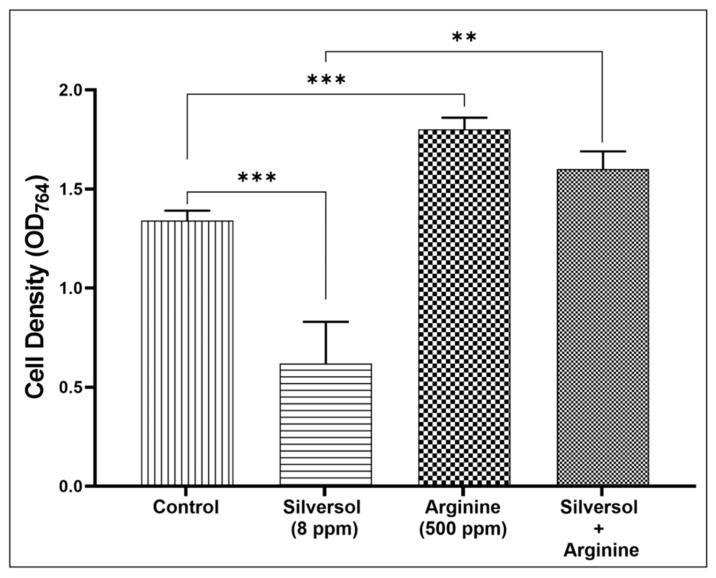
*S. aureus* can resist Silversol’s growth-inhibitory effect in the presence of arginine. When *S. aureus* was challenged with Silversol (sub-MIC) in the absence or presence of arginine, in the latter case Silversol did not demonstrate its antibacterial effect. However, this protection against Silversol conferred on bacteria by arginine-supplementation of media disappeared when a higher concentration of Silversol was used to challenge *S. aureus*. It is possible that at higher concentrations the bactericidal effect of Silversol might stem from additional modes of action, rather than inhibiting arginine biosynthesis ** *p*  ≤  0.01, *** *p* ≤ 0.001.

To provide increased confidence in our finding that one of the major modes of action of Silversol against *S. aureus* is to interfere with arginine synthesis, we conducted an additional experiment wherein *S. aureus* was incubated either in Silversol- or L-arginine (HiMedia)-supplemented media, as well as in a media containing both Silversol and arginine together. Arginine supplementation allowed bacteria to overcome the inhibitory effect of Silversol (Figure 7), confirming Silversol’s ability to interfere with arginine metabolism. This observation becomes relevant in the context that amino acid pathways have recently been targeted as a novel approach to managing bacterial infections. In particular, arginine metabolism has been illustrated to be important for bacterial pathogenesis [114]. Since arginine and its metabolites are utilized as energy sources by various pathogens, its reduced availability can trigger nutrient stress. Reduced availability of arginine may also lead to reduced expression of various pathogenicity genes. The importance of arginine metabolism has been reported in multiple pathogens like *Salmonella typhimurium, Helicobacter pylori, Streptococcus pneumoniae*, *Pseudomonas aeruginosa*, and *Mycobacterium tuberculosis* as a source of energy and as a trigger for polyamine synthesis required for efficient pathogenesis. Owing to the critical role of arginine in establishing pathogenesis, several pathogens employ an array of mechanisms for finetuning arginine metabolism [115], and breach of this finetuning can be expected to compromise bacterial fitness. While pathogens employ strategies to counteract immune responses by interfering with host arginine metabolism, our study shows Silversol to perform the same against *S. aureus*. However, Silversol’s growth-inhibitory action against other pathogens may or may not stem from the same mode of action. For example, in our recent study [37] describing Silversol’s anti-*P. aeruginosa* activity, arginine metabolism was not found to be among the physiological traits attacked by this colloidal silver formulation. It is possible that despite being a broad-spectrum antibacterial agent, the mode of action of Silversol against different pathogenic bacteria may differ more or less.

Arginine has an important role as a substituent for potassium. Under conditions of potassium limitation, bacteria respond by overproduction of arginine, which may partially substitute for potassium to buffer the negative charge of DNA [116]. However, in the presence of Silversol, *S. aureus* seems to have failed to do so, as in our study, the Silversol-exposed *S. aureus* is simultaneously suffering from potassium-limitation as well as down-regulation of arginine biosynthesis.

A gene co-occurrence pattern analysis of gene families across genomes (via STRING) was also conducted with respect to the two potential hubs identified by us and confirmed using PCR (Figure 6A). These two genes appeared to have homologues in a few other important pathogens too (Figure 6B), particularly *Streptococcus pneumoniae*. Whether Silversol’s antibacterial action against other pathogenic bacteria also involves targeting arginine biosynthesis remains an interesting question to be pursued.

## 4. Conclusions

**Figure 8:**
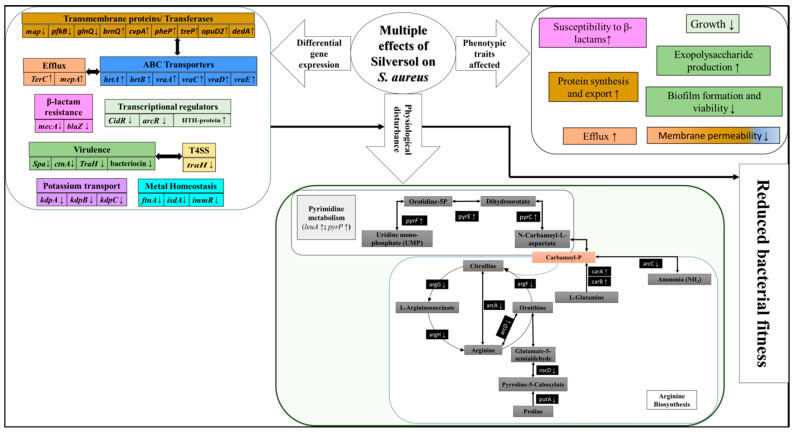
A schematic summary of multiple effects of Silversol on *S. aureus*. Different physiological and phenotypic traits of *S. aureus* affected under the influence of sub-lethal levels of Silversol are shown. Boxes with identical colors have mutual functional relevance. (↑) or (↓) arrow indicates the up- or down-regulation of the concerned gene.

This study investigated the antibacterial effect of the colloidal nanosilver formulation, Silversol, against antibiotic-resistant *S. aureus*. Silversol could exert its antibacterial effect at ppm-level concentration, while it has been shown to be non-toxic at even higher concentrations for cell lines, animals, and humans. A summary of various studies on the safety of Silversol for human use can be read in [23]. This formulation was found to inhibit the growth of *S. aureus* by targeting a wide variety of genes and physiological traits including efflux, biofilm and exopolysaccharide formation, antibiotic susceptibility, arginine biosynthesis, protein synthesis, potassium uptake, transcriptional regulators, etc. A summary of multiple modes of action of Silversol against *S. aureus* is presented in Figure 8. Particularly, arginine metabolism appeared to be one of the major targets of Silversol in *S. aureus*. While aromatic amino acid biosynthesis has been shown as a viable target in *S. aureus* [117], and *argJ* was shown to be a potential core regulator for *S. aureus* persistence in various stresses [94]; to the best of our knowledge, this is the first report showing *argG* and *argH* as important antibacterial targets in this pathogen. Though the antibacterial mechanisms of silver and its nanoformulations have been investigated extensively over the past decades, arginine biosynthesis was hitherto not shown to be targeted by silver in susceptible bacteria. Greater insights into the arginine metabolism of pathogenic bacteria and the relationship of arginine metabolism with bacterial pathogenesis would provide possible targets for controlling bacterial infections. Arginine-tagged drug delivery systems can be designed to target specific subcellular locations like pathogen-containing vacuoles inside host cells. In view of the rampant antibiotic resistance, targeting arginine biosynthesis in bacterial pathogens may be a potent approach toward the development of next-generation anti-pathogenic formulations. Important targets in *S. aureus* attacked by Silversol identified in this study can prove vital input for other antibacterial drug discovery programs. Amino acid biosynthesis pathways are critical for bacterial growth in nutrient-limiting conditions in the host. Surprising connections between bacterial nutrient biosynthesis and antibiotic resistance have been revealed recently [118]. These idiosyncratic connections offer an untapped opportunity for designing novel approaches to combat antibiotic-resistant pathogens.

## Figures and Tables

**Table 1:** Primer sequences for the target genes.

Gene ID/Name	Primers	Amplicon Size (bp)
*argG* (DA471_RS02560)	FP: 5′-TCAAAACCTATGGGGCAGAG-3′	233
	RP: 5′-TTCCGATACCATGCTTACCA-3′	
*argH* (DA471_RS02555)	FP: 5′-TGCAACTATGCTTGCGAATC-3′	189
	RP: 5′-TGCAACTTACCACCAGCATC-3′	
*rpsU* (DA471_RS07645) (Control gene)	FP: 5′-AATGAATCACTTGAAGATGCGTTA-3′	150
	RP: 5′-TTTGAATTTACGTTTACGTGCAG-3′	

**Table 2:** *Staphylococcus aureus* challenged with antibiotics following pre-treatment with Silversol.

Antibiotic	Concentration (µg)	Diameter of Zone of Inhibition (mm)		% Difference
		Control	Experimental	
Penicillin G	10	14 ± 1.14	20.5 ± 2.12	46.42 **
Oxacillin	1	23 ± 7.07	25.5 ± 6.36	Non-significant
Erythromycin	15	R	R	
Clindamycin	2	R	R	
Linezolid	30	29.5 ± 3.53	30 ± 2.12	
Co-trimoxazole	25	35 ± 0	35 ± 0	
Vancomycin	30	20.5 ± 2.12	20 ± 0.70	
Tetracycline	30	29 ± 1.14	31 ± 1.41	
Chloramphenicol	30	28 ± 1.41	28 ± 1.41	
Gentamicin	10	23 ± 12.02	23.5 ± 9.19	
Azithromycin	15	R	R	
Ofloxacin	5	35 ± 0	36 ± 1.41	
Methicillin	5	26.5 ± 9.19	23.5 ± 4.94	
Amoxycillin/clavulanic acid	30	14 ± 0	16 ± 2.82	
Clarithromycin	15	R	R	
Ampicillin	10	14 ± 1.41	21.5 ± 2.12	53.57 *
Amikacin	30	22.5 ± 2.12	23 ± 2.82	Non-significant
Cephalothin	30	30 ± 7.07	31 ± 5.65	
Novobiocin	5	36 ± 1.41	36 ± 1.41	
Teicoplanin	10	21 ± 1.41	21.5 ± 0.70	

An antibiogram of the test strain was generated using the antibiotic discs-Icosa G-I Plus (HiMedia, Mumbai, India) by performing a disc diffusion assay in line with the CLSI guidelines. R: resistant, i.e., no zone of inhibition was observed. * *p* ≤ 0.05, ** *p* ≤ 0.01.

**Table 3:** List of down-regulated genes in Silversol-exposed *S. aureus* satisfying the dual criteria of log fold change ≥ 2 and FDR ≤ 0.05.

Sr. No.	Feature ID	Gene	Product/Function	Log FC	FDR	Node Degree
1	DA471_RS06440	*CidR*	LysR family transcriptional regulator	5.14	1.39 × 10^−6^	NA
2	DA471_RS06435	*leuA*	LeuA family protein	4	0.0003	1
3	DA471_RS09790	*-*	DUF1270 domain-containing protein	3.69	0.0003	NA
4	DA471_RS02560	*argG*	Argininosuccinate synthase	3.28	0.002	7
5	DA471_RS11145	*Spa*	Staphylococcal protein A	3.26	0.002	NA
6	DA471_RS02555	*argH*	Argininosuccinate lyase	3.15	0.002	5
7	DA471_RS02685	*-*	Minor capsid protein	3.08	0.017	NA
8	DA471_RS06430	*tsaC*	L-threonylcarbamoyladenylate synthase	2.99	0.006	NA
9	DA471_RS13755	*arcD*	Arginine–ornithine antiporter	2.96	0.004	4
10	DA471_RS01680	*ctnA*	ABC transporter permease subunit	2.91	0.005	NA
11	DA471_RS11020	*isdA*	LPXTG cell wall anchor domain-containing protein	2.89	0.005	0
12	DA471_RS01245	*-*	Hypothetical protein	2.88	0.005	NA
13	DA471_RS03325	*map*	MAP domain-containing protein	2.84	0.006	0
14	DA471_RS09785	*-*	DUF771 domain-containing protein	2.84	0.006	NA
15	DA471_RS09780	*-*	Hypothetical protein	2.79	0.007	NA
16	DA471_RS09775	*-*	DUF739 family protein	2.78	0.007	NA
17	DA471_RS01255	*traH*	Conjugal transfer protein	2.73	0.008	NA
18	DA471_RS01250	*-*	Hypothetical protein	2.73	0.008	NA
19	DA471_RS05905	*pfkB*	Fructosamine kinase family protein	2.73	0.008	0
20	DA471_RS01275	*-*	Hypothetical protein	2.7	0.009	NA
21	DA471_RS13750	*argF*	Ornithine carbamoyltransferase	2.69	0.009	9
22	DA471_RS13375	*immR*	Helix-turn-helix domain-containing protein	2.66	0.009	0
23	DA471_RS05395	*-*	Hypothetical protein	2.65	0.009	0
24	DA471_RS06160	*kdpB*	K^+^-transporting ATPase subunit B	2.64	0.01	2
25	DA471_RS01260	*RepB*	Replication initiation factor domain-containing protein	2.62	0.01	0
26	DA471_RS06165	*kdpA*	Potassium-transporting ATPase subunit KdpA	2.62	0.01	2
27	DA471_RS13765	*arcR*	Crp/Fnr family transcriptional regulator	2.58	0.01	4
28	DA471_RS06155	*kdpC*	K^+^-transporting ATPase subunit C	2.57	0.01	2
29	DA471_RS13745	*arcA*	Arginine deiminase	2.55	0.01	6
30	DA471_RS00130	*thrS*	Threonine tRNA ligase	2.54	0.01	1
31	DA471_RS13125	*mecA*	PBP2a family beta-lactam-resistant peptidoglycan transpeptidase MecA	2.49	0.02	0
32	DA471_RS11175	*Yxep2*	M20 family metallopeptidase	2.46	0.02	NA
33	DA471_RS13760	*arcC*	Carbamate kinase	2.46	0.02	6
34	DA471_RS10170	*blaZ*	Penicillin-hydrolyzing class A beta-lactamase BlaZ	2.42	0.02	0
35	DA471_RS01675	*glnQ*	Amino acid ABC transporter ATP-binding protein	2.41	0.02	NA
36	DA471_RS08555	*-*	Aminotransferase class I/II-fold pyridoxal phosphate-dependent enzyme	2.41	0.03	NA
37	DA471_RS05580	*ldhD*	D-lactate dehydrogenase	2.34	0.03	NA
38	DA471_RS06060	*nrdG*	Anaerobic ribonucleoside–triphosphate reductase-activating protein	2.32	0.03	0
39	DA471_RS09810	*RecT*	Recombinase RecT	2.32	0.03	NA
40	DA471_RS09795	*-*	DUF1108 family protein	2.3	0.03	NA
41	DA471_RS13995	*-*	Terminase small subunit	2.28	0.03	0
42	DA471_RS09805	*-*	AAA family ATPase	2.27	0.03	0
43	DA471_RS00250	*uspA*	Universal stress protein	2.24	0.04	NA
44	DA471_RS09265	*-*	Lactococcin 972 family bacteriocin	2.19	0.04	NA
45	DA471_RS06295	*fmtB*	LPXTG-anchored DUF1542 repeat protein FmtB	2.19	0.05	NA
46	DA471_RS09840	*-*	Hypothetical protein	2.18	0.05	NA
47	DA471_RS04505	*yghA*	SDR family oxidoreductase	2.17	0.05	NA
48	DA471_RS10350	*ftnA*	H-type ferritin FtnA	2.17	0.05	0
49	DA471_RS01240	*NTPase*	ATP-binding protein	2.15	0.05	0

NA: not applicable, as STIRING did not include these genes in the PPI network.

**Table 4:** List of up-regulated genes in Silversol-treated *S. aureus* fulfilling the dual criteria of log fold change ≥2 and FDR ≤ 0.05.

Sr. No.	Feature ID	Gene	Product/Function	Log FC	FDR	Node Degree
1	DA471_RS04350	-	Hypothetical protein	3.68	0.0003	NA
2	DA471_RS05495	-	FUSC family protein	3.46	0.0007	0
3	DA471_RS00585	-	Proline dehydrogenase	3.45	0.0007	1
4	DA471_RS07140	-	Hypothetical protein	3.43	0.003	NA
5	DA471_RS04685	*hrtB*	ABC transporter permease	3.15	0.002	NA
6	DA471_RS06605	*pyrC*	Dihydroorotase	3.14	0.002	5
7	DA471_RS00725	*-*	Hypothetical protein	3.08	0.003	NA
8	DA471_RS04680	*hrtA*	ABC transporter ATP-binding protein	3.07	0.003	NA
9	DA471_RS06600	*carA*	Carbamoyl phosphate synthase small subunit	2.97	0.004	7
10	DA471_RS11535	*-*	Hypothetical protein	2.93	0.006	NA
11	DA471_RS13920	*cspC*	Cold-shock protein	2.93	0.005	NA
12	DA471_RS06615	*pyrP*	Uracil permease	2.92	0.005	NA
13	DA471_RS06595	*carB*	Carbamoyl-phosphate synthase large subunit	2.83	0.006	8
14	DA471_RS06590	*pyrF*	Orotidine-5′-phosphate decarboxylase	2.78	0.007	4
15	DA471_RS08840	*brnQ*	Branched-chain amino acid transport system II carrier protein	2.69	0.009	0
16	DA471_RS04800	*YbgA*	YbgA family protein	2.68	0.009	NA
17	DA471_RS12105	*CvpA*	CvpA family protein	2.65	0.009	0
18	DA471_RS05490	*-*	DUF2188 domain-containing protein	2.63	0.01	NA
19	DA471_RS11425	*TerC*	TerC family protein	2.63	0.01	NA
20	DA471_RS02540	*rocD*	Ornithine-oxo-acid transaminase	2.63	0.01	3
21	DA471_RS14065	-	Helix-turn-helix domain-containing protein	2.62	0.01	0
22	DA471_RS13410	*vraE*	Peptide resistance ABC transporter permease subunit VraE	2.6	0.01	NA
23	DA471_RS05980	*pheP*	Amino acid permease	2.54	0.01	NA
24	DA471_RS08680	*mepA*	Multidrug efflux MATE transporter MepA	2.46	0.02	0
25	DA471_RS10850	*aldA*	Aldehyde dehydrogenase family protein	2.45	0.02	0
26	DA471_RS05800	*ssaA1*	CHAP domain-containing protein	2.44	0.02	NA
27	DA471_RS06585	*pyrE*	Orotate phosphoribosyltransferase	2.44	0.02	5
28	DA471_RS03355	*-*	Aldo/keto reductase	2.44	0.02	NA
29	DA471_RS13415	*vraD*	Peptide resistance ABC transporter ATP-binding subunit VraD	2.36	0.03	NA
30	DA471_RS12555	*vraC*	Protein VraC	2.33	0.03	NA
31	DA471_RS00355	*serA*	Phosphoglycerate dehydrogenase	2.31	0.03	0
32	DA471_RS00920	*xerC*	Tyrosine recombinase XerC	2.3	0.03	0
33	DA471_RS06690	*treP*	PTS system trehalose-specific EIIBC component	2.3	0.03	0
34	DA471_RS08610	*-*	DUF1398 family protein	2.28	0.03	NA
35	DA471_RS03440	*opuD2*	BCCT family transporter	2.27	0.03	NA
36	DA471_RS10560	*-*	Hypothetical protein	2.25	0.04	NA
37	DA471_RS12545	*vraA*	AMP-binding protein	2.23	0.04	NA
38	DA471_RS13380	*Cspa*	Cold-shock protein	2.22	0.04	0
39	DA471_RS12915	*-*	Hypothetical protein	2.2	0.05	NA
40	DA471_RS02880	*acnA*	Aconitate hydratase AcnA	2.18	0.05	0
41	DA471_RS05505	*DedA*	DedA family protein	2.16	0.05	NA

NA: not applicable, as STIRING did not include these genes in the PPI network. Genes in Table 3 and Table 4 are arranged in descending order of fold change. The databases referred for gene functions are as follows: KEGG (Kyoto Encyclopedia of Genes and Genomes: https://www.genome.jp/kegg/; accessed on 1 January 2024); NCBI gene database (https://www.ncbi.nlm.nih.gov/nuccore/NC_002516; accessed on 1 January 2024); and Uniprot (https://www.uniprot.org/; accessed on 1 January 2024).

**Table 5:** CytoHubba ranking of the top twelve high node degree genes.

No.	Gene ID	Gene Name	Number of Methods Ranking This Protein among the Top 12	Names of 12 Ranking Methods of CytoHubba and Rank Score Provided by Them											
				Degree	MNC	DMNC	MCC	Bottleneck	Eccentricity	Closeness	Radiality	Betweenness	Stress	CC	EPC
1	1200	*carA*	11	8	8	0.466	66	1	0.5	9.5	3.090	9.810	38	0.57	8.39
2	0956	*argG*	10	6	6	0.475	36	1	0.5	8.5	2.909	4.335	22	0.67	7.79
3	0955	*argH*	10	7	7	0.439	42	1	0.5	9	3	9.933	34	0.57	8.21
4	1201	*carB*	10	9	9	0.405	68	2	0.5	10	3.181	20.516	60	0.47	8.49
5	1165	*argF*	9	8	8	0.437	50	5	0.5	9.5	3.090	13.885	44	0.53	8.48
6	1203	*pyrE*	9	5	5	0.518	30	1	0.5	8	2.818	1.598	8	0.8	7.39
7	1199	*pyrC*	3	5	5	0.518	30	1	0.5	8	2.818	1.598	8	0.8	7.40
8	2906	*arcA*	2	5	5	0.324	10	1	0.33	7.833	2.727	4.523	16	0.5	7.45
9	2903	*arcC2*	2	5	5	0.388	12	1	0.5	8	2.818	3.216	14	0.8	7.63
10	2902	*arcR*	2	3	3	0.308	4	1	0.33	6.833	2.545	0.581	4	0.67	5.93
11	1202	*pyrF*	2	4	4	0.568	24	1	0.33	7.166	2.545	0	0	1	6.88
12	0170	*rocD*	2	3	3	0.463	6	1	0.33	6.666	2.454	0	0	1	6.32

*arcD*, one of the high node degree genes, was not considered by cytoHubba.

## Data Availability

All the data are included within the main manuscript or Supplementary Materials.

## Supplementary Materials

The following supporting information can be downloaded at: https://www.mdpi.com/article/10.3390/pharmaceutics16060726/s1, Figure S1: Antibiogram of S. aureus generated through Kirby-Bauer Disc Diffusion assay; Figure S2. Antibiotic-resistant strain of *S. aureus* used in our study could kill all the host worms within 24 h; Figure S3. A schematic presentation of the methodology/workflow employed for whole transcriptome and network analysis; Figure S4. Silversol’s antibacterial effect against *S. aureus* does not follow a strictly linear dose-response pattern; Figure S5. Bacteriostatic and bactericidal effect of Silversol® against *S. aureus* at low and high concentrations respectively; Figure S6. Heat map of DEGs in Silversol®-exposed *S. aureus*; Figure S7. Volcano Plot of experimental versus control samples. Table S1. Antibiogram of antibiotic-resistant *Staphylococcus aureus* generated through Kirby-Bauer Disc Diffusion assay; Table S2. Quantification of extracted RNA, library, and insert size; Table S3. Temperature profile for RT-PCR assay.
